# Does decreased autophagy and dysregulation of LC3A in astrocytes play a role in major depressive disorder?

**DOI:** 10.1038/s41398-023-02665-2

**Published:** 2023-11-25

**Authors:** Shen He, Yue Shi, Jinmei Ye, Jiahui Yin, Yufang Yang, Dan Liu, Ting Shen, Duan Zeng, Min Zhang, Siyuan Li, Feikang Xu, Yiyun Cai, Faming Zhao, Huafang Li, Daihui Peng

**Affiliations:** 1 Shanghai Mental Health Center, Shanghai Jiao Tong University School of Medicine, Shanghai, China; 2 College of Traditional Chinese Medicine, Shandong University of Traditional Chinese Medicine, Jinan, China; 3 School of Integrative Medicine, Shanghai University of Traditional Chinese Medicine, Shanghai, China; 4 Key Laboratory of Environmental Health, Ministry of Education & Ministry of Environmental Protection, School of Public Health, Tongji Medical College, Huazhong University of Science and Technology, Wuhan, China

**Keywords:** Depression, Diagnostic markers

## Abstract

Astrocytic dysfunction contributes to the molecular pathogenesis of major depressive disorder (MDD). However, the astrocytic subtype that mainly contributes to MDD etiology and whether dysregulated autophagy in astrocytes is associated with MDD remain unknown. Using a single-nucleus RNA sequencing (snRNA-seq) atlas, three astrocyte subtypes were identified in MDD, while C2 State-1Q astrocytes showed aberrant changes in both cell proportion and most differentially expressed genes compared with other subtypes. Moreover, autophagy pathways were commonly inhibited in astrocytes in the prefrontal cortices (PFCs) of patients with MDD, especially in C2 State-1Q astrocytes. Furthermore, by integrating snRNA-seq and bulk transcriptomic data, we found significant reductions in LC3A expression levels in the PFC region of CUMS-induced depressed mice, as well as in postmortem PFC tissues and peripheral blood samples from patients with MDD. These results were further validated by qPCR using whole-blood samples from patients with MDD and healthy controls. Finally, LC3A expression in the whole blood of patients with MDD was negatively associated with the severity of depressive symptoms. Overall, our results underscore autophagy inhibition in PFC astrocytes as a common molecular characteristic in MDD and might reveal a novel potential diagnostic marker LC3A.

## Introduction

Currently, major depressive disorder (MDD) affects more than 300 million people worldwide, constituting a global public health problem. A report by the World Health Organization predicted that MDD may become the leading cause of disability globally by 2030 [1]. However, the pathological mechanisms of this devastating disorder remain unclear, and current diagnosis is solely based on behavioral traits and assessment tools. Clinicians face the dilemma of a paucity of objective biomarkers to assist in diagnosing, treating, and monitoring MDD [2]. Therefore, further analysis of the molecular basis of MDD and identification of effective biomarkers are paramount for supporting diagnosis.

Autophagy is a vastly conserved phenomenon in eukaryotic cells. During autophagy, autophagosomes are formed by incorporating components that are degraded into single- or double-layer membrane vesicles. Lysosomes are further fused with autophagosomes to form autolysosomes [3]. Autophagy degrades and recycles damaged organelles and misfolded proteins [4]. Through cellular waste removal and cytosolic constituent delivery, autophagy plays an important role in maintaining cellular homeostasis and regulating the inflammatory response [5]. Recently, the delicate association between autophagy and the pathological mechanisms of MDD has received increased attention. Several studies have reported abnormal expression levels of autophagy-related genes and a deficiency in autophagy in the brains of mice with chronic restraint stress, chronic unpredictable mild stress (CUMS), and LPS-induced depression [6–8]. In addition, research suggests that certain antidepressants may exert their effects by modulating the autophagy pathway [9–11]. Notably, studies have demonstrated that enhancing autophagy through sphingolipids or the mTOR inhibitor rapamycin can alleviate depressive symptoms in mice, while inhibiting beclin-1 is necessary to counteract the effects of antidepressants or antidepressant candidates [12–14]. Furthermore, in human studies, replicated evidence has indicated that autophagy-related genes and proteins hold potential as diagnostic and therapeutic biomarkers for MDD [15–17]. In a previous study, we identified GPR8 as a potential diagnostic biomarker, given its association with autophagy [15]. In addition, our findings suggest that baseline serum beclin-1 levels show promise as a predictive biomarker for antidepressant response in patients with MDD [16]. These findings suggest that impaired autophagy may not only contribute to the pathogenesis of depression but also represent a potential novel therapeutic strategy for treating depression by targeting the autophagy pathway.

Astrocytes are the most abundant among all cell types in the central nervous system (CNS), serving multiple functions, including the maintenance of cellular homeostasis, neurotransmitter recycling, cell viability promotion, and immune signaling [18]. Recent research has emphasized the function of astrocytes in MDD. Various studies have shown that the total number of astrocytes is drastically reduced in the postmortem brains of patients with MDD and in animal models of chronic stress [19–21]. Astroglial markers, including glial fibrillary acidic protein (GFAP) and S100 calcium-binding protein B (S100B), are also decreased in specific cortical and subcortical brain areas [22, 23].

Interestingly, one animal model study reported that fluoxetine exerted a proautophagic effect in primary cultured astrocytes from CMS mice [11]. However, the role of autophagy in astrocyte function in MDD remains poorly understood, with minimal evidence having been obtained from patients with MDD. In addition, it is worth noting that due to the use of bulk homogenates or tissue of postmortem human brains, gene expression changes within individual cell types are usually nebulous in previous molecular studies of MDD [24, 25]. Single-cell RNA sequencing (scRNA-seq) and single-nucleus RNA sequencing (snRNA-seq) technologies, which involve the measurement of genomics, epigenomics, and transcriptomics in individual cells without cell purification, have been rapidly developed in recent years [26]. These technologies can be utilized to explore the biological functions of cell types to facilitate the study of astrocytes. In addition, the prefrontal cortex (PFC) is intimately linked to MDD pathogenesis, and the association between astrocytes in the PFC and MDD has been reported by numerous studies [27–29]. However, the role of autophagy in astrocytes in the PFCs of patients with MDD remains unclear.

In this study, by combining snRNA-seq data with bulk transcriptomic data, we identified a reduced proportion of astrocytes in the PFC of MDD patients. We then explored communication between astrocytes and other cell types in the PFC. Moreover, we further demonstrated that, in astrocytes, the activity of autophagy-related pathways was significantly inhibited, and MAP1LC3A (LC3A) was downregulated in MDD brains. Furthermore, transcriptome profiling of peripheral blood from patients with MDD showed LC3A downregulation. Finally, we validated the significant downregulation of LC3A expression by real-time polymerase chain reaction (quantitative PCR, qPCR). Taken together, these findings suggest for the first time that LC3A may be a potential diagnostic biomarker of MDD and provide new insights into the pathogenesis of MDD.

## Materials and methods

### Processing of snRNA-seq data from PFCs of patients with MDD

First, the snRNA-seq expression matrix for GSE144136 was acquired from the Gene Expression Omnibus (GEO, https://www.ncbi.nlm.nih.gov). Currently, this is the only publicly available single-cell dataset from postmortem PFCs of patients with MDD, and raw gene barcode matrices were processed by the original authors [26]. They processed the original data and excluded high dropout genes, potential doublets, and low-quality cells, retaining a total of 30,062 genes and 78,886 cells. Based on the downloaded data, the top 2000 highly variable genes were identified using the FindVariableFeatures function in the Seurat R package (v.4.1.0) [30]. Then, the data were normalized and converted to a log scale using the ScaleData function. We also performed cell cycle scoring analysis using the CellCycleScoring function in the Seurat R package but did not find any effects of the cell cycle on clustering. Therefore, cell cycle genes were retained, and the cell cycle did not regress. We subsequently applied the RunPCA function with npcs = 50 to propose a principal component analysis algorithm. The first 30 principal components were maintained. For visualization, the RunUMAP function with default parameters was used to calculate the uniform manifold approximation and projection (UMAP). Then, to obtain biologically significant clusters, we constructed a shared nearest neighbor (SNN) graph using the FindNeighbors function with a resolution of 1. For cell-type annotation, cell identity was automatically assigned using CellTypist [31], which uses stochastic gradient descent learning to dissect and annotate cell populations using logistic regression. To aid the assignment of cell types, manual annotation based on marker genes [32, 33] was performed. We then utilized the CellChat R package (version.1.6.0) to assess potential ligand–receptor interactions or cell–cell interactions [34]. Genes with a log-fold change greater than 0.1( | logFC | ) and *P* value less than 0.05 (*P* < 0.05) were identified as differentially expressed genes (DEGs) using the FindMarkers function. Based on the astrocyte subset of the Seurat subject, gene set enrichment analysis (GSEA) [35] was performed to detect the biological function of astrocytes. The top 20 pathways were obtained, including autophagy-related pathways. To further identify the activity of the autophagy pathway, we performed AUCell [36] enrichment analysis to define the pathway activity of individual cells. Autophagy-related gene sets included the chaperone-mediated autophagy, selective autophagy, and autophagy pathways, which were obtained from the Reactome database (https://reactome.org/).

To subcluster astrocytes from MDD samples, harmony alignment [37] was performed to eliminate intersample variation. The top 20 harmony dimensions were used to cluster the subset and perform the UMAP analysis. The dimensional reduction process yielded three astrocyte subpopulations. The default settings of COSG [38] were then applied to identify differentially expressed genes in each subgroup. In reference to a previous study [39], manual annotation was performed to facilitate astrocyte subclustering.

Pseudotime analyses were performed with the Monocle 3R package (https://cole-trapnell-lab.github.io/monocle3/) to construct evolutionary trajectories of the three subgroups of astrocytes [40]. To evaluate the transcription factor (TF) analysis regulation strength, we followed the single-cell regulatory network inference and clustering (PySCENIC, v.0.10.0) [36] workflow according to the demonstration notebook. Next, the LogFC of each TF activity was calculated, and the top six TFs for each cell type are shown.

### Analysis of bulk transcriptomic data from postmortem human brains

To further investigate astrocyte dysfunction, multiple bulk transcriptomic datasets from the postmortem brains of patients with MDD were downloaded and analyzed. We obtained the expression matrices of GSE45642, GSE53987, and GSE92538 from the GEO database. These datasets contained expression profiles from the PFC of patients with MDD and healthy controls. Based on the snRNA-seq data analysis, we obtained the top 300 DEGs in astrocytes compared to other cell types as astrocyte-specific gene signatures. We then calculated single-sample GSEA (ssGSEA) scores for samples from patients with MDD and healthy controls. However, considering that the microarrays of GSE45642 and GSE92538 only detected a limited number of genes, covering only 11875 and 10241 probes, respectively, we only conducted subsequent analyses on the DEGs of GSE53987. Genes with a log-fold change greater than 0.1 ( | logFC | > 0.1) and *P* value less than 0.05 (*P* < 0.05) were identified as DEGs [41].

### Animals

Experimental wild-type C57BL/6J male mice (7–8 weeks old, 22–26 g; GemPharmatech) were used in this study. All animals were group-housed under standard conditions on a 12-h light: dark cycle (12 h light/dark cycle) with ad libitum access to food and water. Animal housing and experimental rooms were maintained at a temperature of 23 ± 1 °C and humidity of 40%. Experiments were conducted during the daytime. All experimental procedures adhered to international guidelines and were approved by the Experimental Animal Committee of Shanghai Jiao Tong University School of Medicine.

### Chronic unpredictable mild stress

Chronic unpredictable mild stress (CUMS) was conducted with modifications as described in previous studies [42, 43]. Briefly, male wild-type mice were randomly assigned to either the control group (*n* = 10) or CUMS group (*n* = 15) in accordance with a random number table. The CUMS group underwent continuous and random exposure to various unpredictable mild stressors over eight weeks, including 6-h restraint, 7-h cage tilt, 30-min air puff, 1.5-min tail pinch, 24-h wet bedding, 24-h food deprivation, 24-h water deprivation, and 24-h light–dark reversal. The control group was housed under standard conditions and handled daily in the animal housing room.

### Behavioral tests

#### Sucrose preference test (SPT)

After completing CUMS, each mouse was individually housed for a two-bottle choice test. During the habituation phase, mice were provided with two bottles of water for 24 h, followed by two bottles of a 1% sucrose solution for another 24-h period. After a 2-day habituation period, mice were deprived of water for 24 h. During the testing phase, each mouse was given access to two bottles containing either water or a 1% sucrose solution, and the amount of liquid consumed over a 24-h period was recorded. To prevent potential side preference by the mice, the positions of the bottles were switched in the middle of the test. The sucrose preference index was defined as the percentage of sucrose solution consumed relative to the total intake of water and sucrose.

#### Forced swimming test (FST)

Mice were placed in glass cylinders (25 ± 1 °C; 20-cm diameter, 30 cm height) filled with water up to 15 cm for a duration of 6 min. Video recordings were taken during the entire six-minute duration, excluding the first 2 min of the forced swim test. The time spent immobile was determined by two observers who were blinded to the experimental conditions. Immobility was defined as the absence of any active movement except for that necessary to maintain the animal’s head above the water.

#### Open field test (OFT)

To acclimate each mouse to the testing environment, they were placed in the experimental room 2 h prior to the behavioral test. During the test, each animal was transferred to the central zone of an open field chamber (40 × 40 cm^2^) under dim light for 15 min. The behavior of each mouse was recorded using Topscan, a video tracking system developed by CleverSys, and analyzed accordingly. Anxiety-like behavior was evaluated based on the distance traveled and time spent in the central zone, while locomotion was assessed by measuring the total distance traveled within the open field. All the above behavioral tests were performed by the same experimenter blinded to the group assignment to avoid bias.

### Western blot

Upon isoflurane anesthesia, the brains of sacrificed mice were quickly removed and placed on ice. The medial prefrontal cortex (mPFC) was then rapidly dissected from the brain tissue. For protein sample preparation, brain tissues were homogenized in M-PER Mammalian Protein Extraction Reagent (Thermo Fisher Scientific, 78501), and supernatants were collected after centrifugation at 10,000×*g* for 15 min at 4 °C. Following protein extraction, the concentrations of protein samples were determined using the bicinchoninic acid (BCA) assay (Thermo Fisher Scientific, 23225). Equal amounts of protein samples were loaded and separated on 15% SDS-PAGE gels. Primary antibodies against LC3A (Proteintech, 18722-1-AP, 1:1000) and UBE2N (GeneTex, GTX113290, 1:1000) were used, along with an anti-rabbit secondary antibody β-Actin (Bioworld Technology, AP0060, 1:5000). Band intensities were quantified using ImageJ software (NIH).

### Participants

Group 1 included 19 patients with MDD and 17 healthy controls from the Shanghai Mental Health Center (Supplementary Table 1). Group 2 included 33 patients with MDD and 32 healthy controls from the Shanghai Mental Health Center (Supplementary Table 2). All procedures were reviewed and approved by the ethical committee of Shanghai Mental Health Center. The ethics number is 2018–39. The study was conducted according to the Declaration of Helsinki and written informed consent was obtained from all participants.

The inclusion criteria were as follows: (1) MDD diagnosed according to the Diagnostic and Statistical Manual of Mental Disorders-IV (DSM-IV) diagnostic criteria; (2) 18–65 years of age; (3) Han ethnicity; and (4) patients who had not taken any antidepressants within 2 weeks prior to enrollment. Participants who met the following criteria were excluded: (1) having any psychiatric disorders other than MDD; (2) having any serious physical disease (i.e., organic brain disease, autoimmune disease, cardiovascular disease, and cancer); (3) being pregnant or breastfeeding; (4) alcohol or other substance abuse; and (5) having received MECT and rTMS within 8 weeks prior to enrollment. Clinical symptoms were evaluated using the Hamilton Depression Rating Scale (HAMD)-17. Healthy controls with a family history of mental illness were excluded.

### Sample preparation

Peripheral whole blood (2.5 mL) was drawn from all participants and collected in PaxGene Blood tubes. The samples were stored at −80 °C before collection was complete. The PaxGene isolation kit (Qiagen) was used to isolate total RNA, according to the manufacturer’s instructions. RNA purity and concentration were evaluated using a NanoDrop ND-1000 (Thermo Scientific, Waltham, MA, USA) for RNA sequencing and RT-PCR analyses.

### Library preparation and RNA sequencing analysis

Whole-blood samples from 19 patients with MDD and 17 healthy controls from Study 1 were used for bulk RNA sequencing analysis. RNA-Seq libraries were established based on 1 µg of total RNA using a KAPA Stranded RNA-Seq Kit. RNA-seq was performed on an Illumina Nova Seq 6000 sequencer (Illumina, San Diego, CA, USA). Solexa pipeline v1.8 was used to conduct image analysis and base calling. Sequence quality was checked using FastQC software. The trimmed reads were ligated to the human reference genome version 19 using Hisat2 software. The DESeq2 algorithm was used to perform differential gene expression analysis on the counts. Genes with a *P* value < 0.05 and |log-fold change| ( | logFC | ) ≥ 0.1 were considered DEGs [41]. KEGG enrichment analysis was conducted using the R package clusterProfiler (v3.18.1), and the top 20 enriched signaling pathways were obtained.

### PCR analysis

Whole-blood samples from 33 MDD patients and 32 healthy controls from Study 2 were used for qPCR analysis. To measure LC3A (assay ID: Hs01076567_g1), cDNA was first synthesized and then amplified by qPCR using the Thermo Scientific RevertAid First Strand cDNA Synthesis Kit. Real-time quantitative reverse transcriptase-PCR was performed on an Applied Biosystems™ QuantStudio™ 5 Dx Real-Time PCR Instrument with a 20 μL PCR mixture that contained 4 μL of RT product, 1 μL of 20× TaqMan Gene Expression assay, 10 μL of 2× TaqMan Universal PCR Master Mix, with UNG (Applied Biosystems, Foster City, CA, USA) and 5 μL of nuclease-free water. PCRs were run at 50 °C for 2 min and at 95 °C for 10 min, followed by 95 °C for 15 s and at 60 °C for 1 min in 384-well plates. GAPDH (assay ID: Hs99999905_m1) was used as the endogenous control gene. Relative gene expression was calculated using the 2^−ΔΔCT^ method [44].

### Statistical analysis

Chi-square tests were used to evaluate categorical variables. The normality test was conducted using the Shapiro–Wilk test. The Mann*–*Whitney *U* test was used to analyze differences in LC3A expression levels between patients and healthy participants. To explore the diagnostic potential of LC3A, we performed receiver operating characteristic (ROC) curve analysis. All results are presented as the mean ± standard deviation (SD). Statistical significance was defined as *P* < 0.05 (two-tailed). R statistical software (v.4.1.0) was used for bioinformatics analysis. Other statistical analyses were conducted using SPSS software (version 23.0).

## Results

### Decreased proportion of astrocytes in the PFC in MDD

**Fig. 1:**
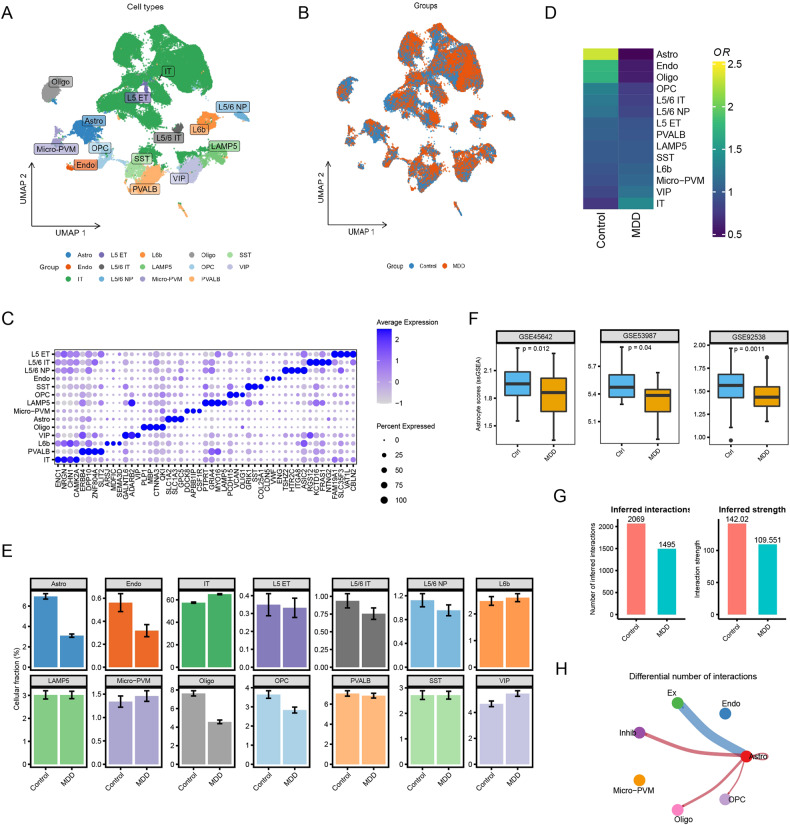
Single-cell gene expression profiling of PFC derived from postmortem brains. **A** Uniform manifold approximation and projection (UMAP) map of 78,886 cells from 34 donors. Each dot represents one cell, colored according to the indicted cell types. Astro astrocytes, L5 ET layer 5 extratelencephalic-projecting neurons, L6b layer 6b neurons, Oligo oligodendrocytes, SST somatostatin-positive GABA neurons, Endo endothelial cells, L5/6 IT layer 5/6 intratelencephalic cells, OPC oligodendrocyte precursor cells, VIP vasoactive intestinal peptide-positive GABA neurons, IT intratelencephalic cells, L5/6 NP layer 5/6 near-projecting neurons, Micro-PVM microglial cells and perivascular macrophages. **B** Visualization of clustering results colored according to groups. **C** Average expression levels of marker genes in different cell clusters. **D**, **E** Heatmap and bar plot showing the odds ratios (**D**) and proportions (**E**) of the 14 major cell types in MDD and control brains. **F** Box plot showing the reduced proportion of PFC astrocytes in three bulk expression profiling datasets. **G** Bar plot showing the inferred interactions and inferred strength of cell–cell communication in each group. **H** Circle plot showing cell–cell communication focusing on astrocytes. Edge width represents the communication score.

We first performed snRNA-seq analysis of postmortem brain areas of the PFC from 17 patients with MDD and 17 controls (see “Methods”). A total of 14 cell types were identified in the combination analysis of MDD patients and healthy controls using previously reported cell-type markers [32, 33] (Fig. 1A–C). We subsequently compared the proportions of different cell clusters between patients with MDD and controls. Using the expected cell numbers with odds ratio (OR) analysis, astrocytes showed a strong distribution preference in the healthy control group (Fig. 1D). Consistently, bar plots showed that the proportion of astrocytes was reduced in the MDD samples compared to that in the control samples (Fig. 1E). This was further validated by three bulk human MDD PFC transcriptome cohorts, GSE45642, GSE53987, and GSE92538 (Fig. 1F). Together, these findings indicate that astrocytes may be crucial for the pathophysiology of MDD.

### Decreased intercommunication between astrocytes and other cell types

To explore the intercommunication between reactive astrocytes and other cell clusters in the CNS, we performed cell–cell communication analysis, which revealed attenuated interaction and weakened interaction strength across all cell clusters in MDD brain samples compared to control brain samples (Fig. 1G). Focusing on astrocytes, we found that the intercommunication between astrocytes and excitatory neurons decreased, while cell–cell communication between astrocytes and inhibitory neurons, oligodendrocytes, and oligodendrocyte precursor cells (OPCs) was enhanced (Fig. 1H). These results further demonstrated that astrocytes are closely linked to the pathology of MDD.

### Autophagy pathway inhibition in astrocytes may be associated with MDD pathology

**Fig. 2:**
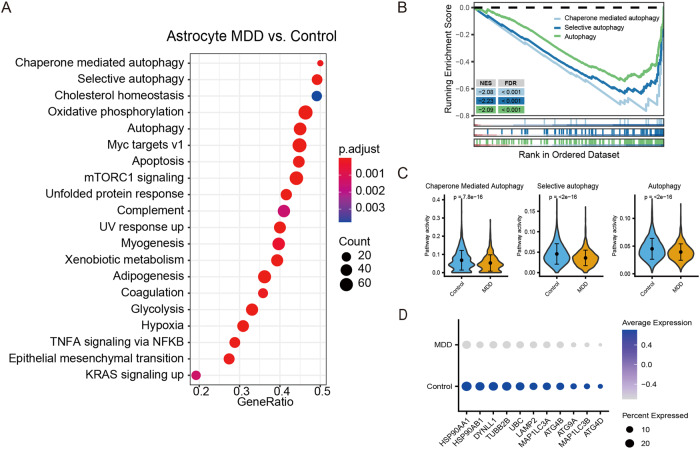
Pathway enrichment analysis of astrocytes indicating the inhibition of autophagy pathways in patients with MDD. **A** GSEA pathway enrichment analysis of differentially expressed genes (DEGs) in MDD versus control subjects in astrocytes. **B** Enrichment plot showing the inhibition of the chaperone-mediated autophagy pathway, selective autophagy pathway, and autophagy pathway. **C** AUCell analysis showing the inhibition of autophagy pathways in MDD. **D** Dot plot showing the reduced expression levels of autophagy-related genes in MDD.

To obtain more detailed analyses of biological function, we applied the GSEA algorithm for pathway enrichment analysis of astrocytes in patients with MDD. The results revealed that several autophagy-related signaling pathways, including chaperone-mediated autophagy, selective autophagy, and autophagy, were enriched in astrocytes from patients with MDD (Fig. 2A). All autophagy-related pathways were significantly inhibited (Fig. 2B). These findings are consistent with those of previous studies based on animal models [11, 45, 46] and can be further confirmed using AUCell analysis (Fig. 2C) to quantify the activity of predicted regulons. In addition, some of the autophagy-related genes were significantly downregulated in the MDD group, namely, LAMP2, MAP1LC3A (LC3A), ATG4B, ATG9A, MAP1LC3B, and ATG4D (Fig. 2D).

Major depressive disorder is not only a disease of astrocytes; neurons also play an important role in the pathological mechanisms of MDD [47, 48]. Thus, we investigated autophagy regulation in all types of neuronal cells and found that significant changes in autophagy-related pathway activity existed in some neurons (Supplementary Fig. 1A), which suggests that autophagy plays a wide functional role in both astrocytes and neuronal cells. However, unlike astrocytes, GSEA enrichment analysis demonstrated that autophagy-related pathways did not rank among the top enriched pathways in neurons (Supplementary Fig. 1B). Therefore, this study mainly explored autophagy in astrocytes. In future studies, we hope to further explore autophagy in neurons.

### The activity of autophagy-related pathways decreased most significantly in the C2 subpopulation of astrocytes

**Fig. 3:**
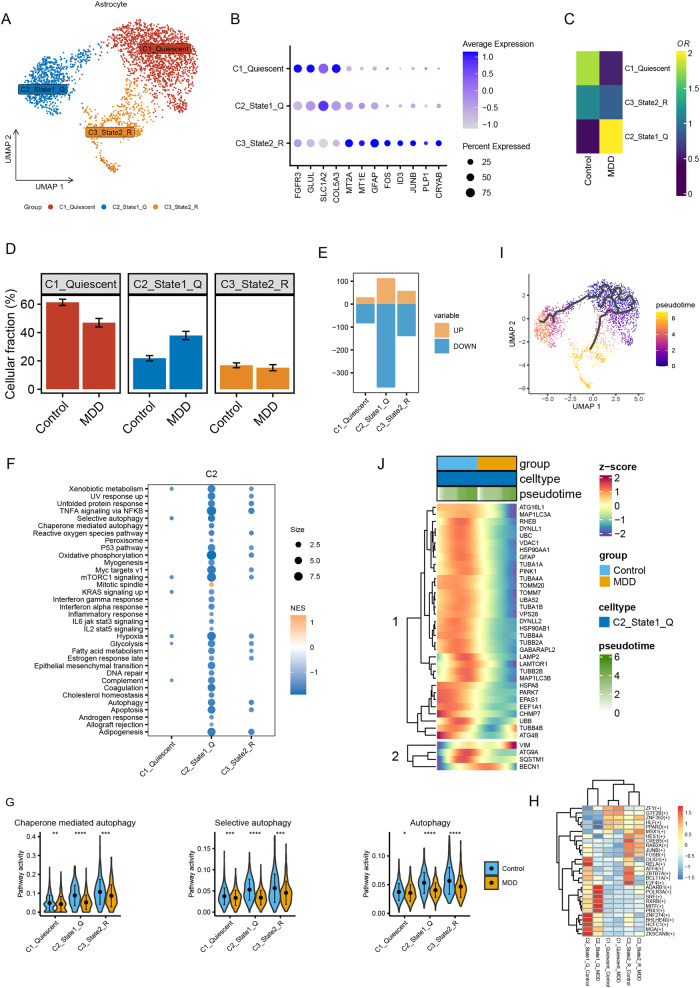
C2 The State-1-Q astrocyte subpopulation is associated with inhibition of autophagy-related pathways in the MDD PFC. **A** UMAP showing the distributions of three astrocyte subpopulations. **B** Dot plot showing the expression level of genes with subpopulation-specific patterns. The dot size indicates the proportion of cells in each subpopulation. **C**, **D** Heatmap and bar plot showing the odds ratios (**C**) and proportions (**D**) of the three subpopulations in MDD and control brains. **E** Bar plot showing the number of DEGs in each subpopulation. Yellow bars indicate upregulated genes and blue bars indicate downregulated genes. **F** GSEA in brains from patients with MDD compared to control subjects in different astrocyte subpopulations. **G** AUCell analysis showing the inhibition of autophagy pathways in astrocyte subpopulations. **H** Heatmap revealing the TF activities in astrocyte subpopulations. **I** Pseudotime trajectory of astrocytes. **J** Heatmap showing the alteration in the expression of autophagy-related genes during astrocyte differentiation.

Although astrocytes are essential in MDD pathology, their subtypes have been poorly explored. Based on the biomarkers of subpopulations reported in previous studies [39], astrocytes were further classified into three subpopulations: C1 quiescent, C2 state-1 quiescent (State-1Q), and C3 state-2 reactive (State-2R) astrocytes (Fig. 3A, B). C1 astrocytes had low levels of MT2A, low GFAP, and high SLC1A2 levels, representing a quiescent state. C2 astrocytes with high levels of MT2A, low GFAP, and high SLC1A2 were classified as C2 state-1Q, indicating quiescent astrocytes with early reactive features. C3 astrocytes with high levels of MT2A, high GFAP, and low SLC1A2 were classified as state-2R, denoting reactive astrocytes with high expression of metallothionein-related genes.

Among the three subtypes, the proportion of C2 was higher in MDD brain samples than in control subjects, whereas the proportions of C1 and C3 were both lower in the MDD group (Fig. 3C, D). Intriguingly, C2 had the highest number of DEGs (Fig. 3E), suggesting the most heterogeneous state in C2 astrocytes between MDD and healthy controls compared to other subtypes. We subsequently performed GSEA on the three subtypes and found that the enrichment pathways of C2 astrocytes were the most abundant (Fig. 3F), indicating that C2 state-1Q astrocytes in the PFC were closely linked to MDD. More importantly, autophagy-related pathways in C2 were more commonly inhibited than those in other subpopulations (Fig. 3F). Further analysis using the AUCell method also showed that, compared to the C1 and C3 subpopulations, signaling pathways of chaperone-mediated autophagy, selective autophagy, and autophagy were consistently significantly decreased in the C2 subpopulation of astrocytes (Fig. 3G). These results indicate that the autophagy pathway in C2 State-1Q astrocytes in the PFC is closely linked to MDD. Moreover, we used pySCENIC analysis to explore TF activities that characterize these specific cell states. The results showed differential activities of various TFs in different subpopulations of astrocytes (Fig. 3H). Some transcription factors, such as ATF4 and BHLHE40, have been found to regulate the autophagy pathway and are associated with the pathological mechanism of MDD [49–51].

### Autophagy-related genes in C2 state-1Q subpopulations are progressively downregulated during differentiation

To further explore the molecular mechanisms driving astrocyte heterogeneity and the differentiation relationship between individual subpopulations in MDD, we performed pseudotime subclustering and differentiation trajectory analysis of astrocytes using Monocle3. Three cell branches with distinct differentiation patterns were identified (Fig. 3H). Consistent with a previous work [39], the cells distributed in C1 were supposed to be the initial cell type and further differentiated into C2 and C3 cell types (Fig. 3I). During differentiation, autophagy-related genes in C2 followed a decrease in the expression level along the trajectory, such as ATG16L and LC3A (Fig. 3J), suggesting that this trajectory described the autophagy-related transcriptional changes during the transition from a homeostatic to a disease-associated cell state, which further demonstrated that autophagic changes in astrocytes in the PFC were associated with the pathology of MDD.

### LC3A may be associated with MDD pathogenesis

**Fig. 4:**
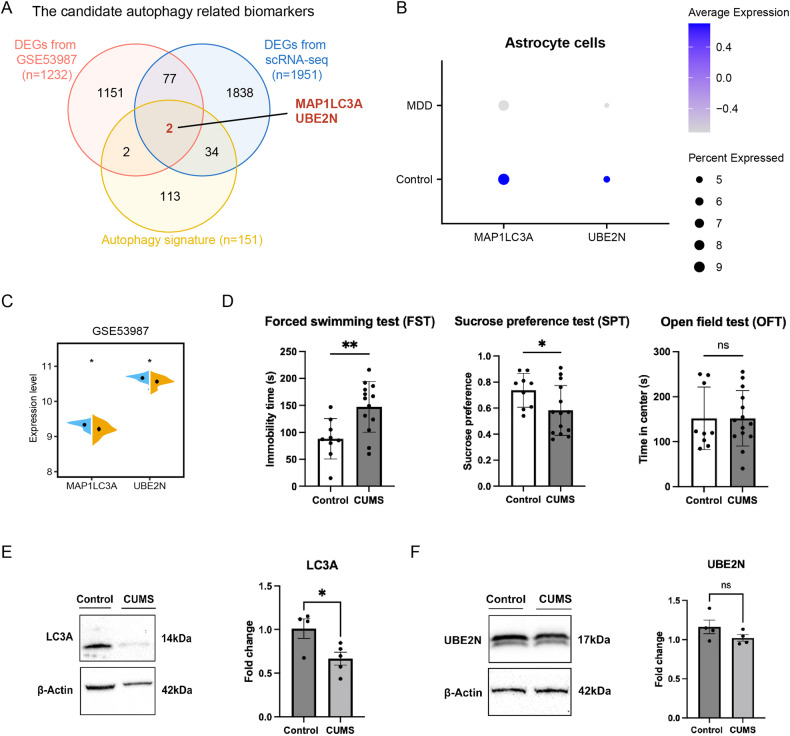
Downregulation of LC3A in the PFC from MDD patients and CUMS mice. **A** Venn diagram of DEGs from the GSE53987 bulk transcriptomic dataset, DEGs from snRNA-seq data and autophagy-related signature genes. Numbers in colored areas represent the gene numbers in the corresponding overlap. MAP1LC3A and UBE2N are the shared DEGs of the three gene lists. **B** Dot plot showing the expression levels of LC3A and UBE2N in astrocytes from samples of MDD and control subjects. **C** The expression levels of LC3A and UBE2N in the prefrontal cortex from subjects with MDD and the control group in the GSE53987 dataset. **D** Time spent immobile in the FST; Sucrose consumption (%) in the SPT; OFT time spent in the center area. All data are expressed as the mean ± SD. *P < 0.05, compared with control group. **E** Detection of LC3A in the PFC of each group by Western blot analysis. All data are expressed as the mean ± SD. *P < 0.05, compared with the control group. **F** Detection of UBE2N in the PFC of each group by Western blot analysis. All data are expressed as the mean ± SD. *P < 0.05, compared with the control group. ns not significant.

Given the crucial role of autophagic pathways in PFC astrocytes, we further developed an integrative procedure by combining bulk and snRNA-seq transcriptomic data to explore the key autophagy-related marker genes in MDD, which could effectively balance the strengths and weaknesses of the individual bulk and snRNA-seq technologies to identify stable biomarkers or therapeutic targets involved in MDD. To do so, we first generated a reference gene list of autophagy by extracting all genes of the three autophagy-related pathways, obtaining 151 autophagy-related genes. Moreover, a total of 36 genes were found to overlap between the reference gene list and DEGs of PFC astrocytes between patients with MDD and healthy controls (Fig. 4A). Further analysis showed that LC3A and UBE2N shared DEGs in the bulk transcriptomic dataset GSE53987 and 36 overlapping genes (Fig. 4A). Downregulation of LC3A and UBE2N was observed in the prefrontal cortex (PFC) brain region and in astrocytes located in the PFC of patients with MDD (Fig. 4B, C).

In addition, we established a CUMS mouse model and used Western blot analysis to measure the expression levels of LC3A and UBE2N in the PFC region of the brain (Fig. 4D–F). Our results revealed that LC3A expression was significantly decreased in the PFC of CUMS mice compared to the control group (Fig. 4E). However, there was no significant change in UBE2N expression between the two groups (Fig. 4F).

### LC3A may be a potential diagnostic biomarker for MDD

**Fig. 5:**
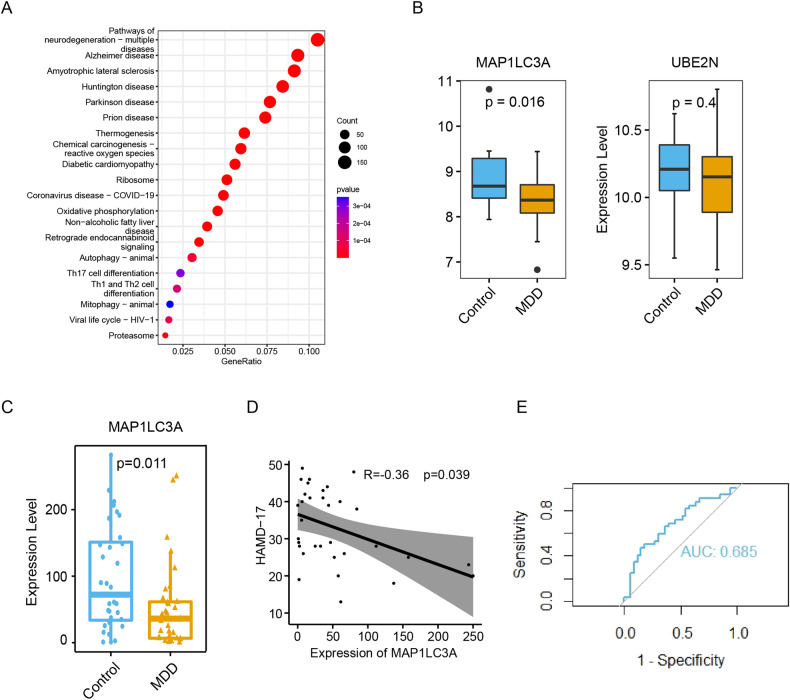
Downregulation of LC3A in MDD in peripheral blood. **A** KEGG enrichment analysis of DEGs from RNA-seq transcriptome profiling of peripheral blood. **B** Box plot showing the expression levels of LC3A and UBE2N between MDD and healthy controls based on RNA-seq data of peripheral blood. **C** Violin plot showing the expression level of LC3A in peripheral blood samples from MDD patients and healthy controls by qPCR. **D** Scatter plots showing Spearman correlation between the LC3A expression level in the peripheral blood samples and corresponding HAMD-17 total scores. **E** ROC analysis for LC3A between patients with MDD and healthy control subjects in PCR data.

To further investigate whether LC3A and UBE2N could be used as diagnostic biomarkers of MDD, we first conducted a preliminary exploration of differential expression of the autophagy-related genes LC3A and UBE2N in the peripheral blood from 19 MDD patients and 17 healthy controls using RNA-seq. This allowed us to investigate the signaling pathways enriched by the entire set of DEGs simultaneously. Interestingly, DEGs in peripheral blood samples were also enriched in autophagy-related pathways, indicating a role for autophagy in MDD (Fig. 5A). Compared to the control samples, LC3A was significantly downregulated in MDD samples, while the expression level of UBE2N failed to show a significant change (Fig. 5B). However, due to the presence of inherent sequencing noise and library construction bias associated with RNA-Seq, we validated our findings independently using qPCR in a separate cohort of patients. We measured the expression levels of LC3A in peripheral blood samples from 33 MDD patients and 32 healthy controls using qPCR. The results also demonstrated that LC3A was significantly lower in MDD and negatively correlated with the severity of MDD symptoms (Fig. 5C, D).

As the snRNA-seq data used in our study were obtained from the brain tissue of 17 male control subjects and 17 male MDD patients, there exists a significant disconnect between the gender of these data and the participants included in our investigation. To address this point, we conducted separate analyses of the RNA-Seq data for males and females. Our results showed that male subjects exhibited a significant reduction in LC3A expression while female subjects displayed a decreasing trend. In contrast, no significant changes in UBE2N expression were observed in either male or female patients when compared to healthy controls of the same gender (Supplementary Fig. 2). Nevertheless, given the small sample size of our sequencing data, we took further measures to mitigate this gender discrepancy by utilizing PCR data. Although we had a limited number of male samples in our PCR analysis, we focused our investigations solely on female samples. Our findings indicated a significant reduction in LC3A expression among female MDD subjects compared to healthy female controls (Supplementary Fig. 3). Ultimately, our study suggests that LC3A expression may be significantly reduced in MDD patients and may not be gender-dependent. To further investigate the diagnostic potential of LC3A for MDD, we performed ROC analysis on both RNA-Seq and PCR data. Our results indicated a moderate diagnostic value of LC3A for MDD in both datasets with areas under the curve (AUCs) of 0.659 and 0.685, respectively (Supplementary Fig. 4 and Fig. 5E).

## Discussion

Major depressive disorder (MDD) is a highly heterogeneous disorder. Previous bulk RNA-seq methods have limited researchers to decoding brain cell-type-specific gene expression patterns and exploring the relationship between specific cell types and the pathogenesis of MDD. Thus, single-cell sequencing could help us better understand this key point.

In this study, we analyzed a published large-scale snRNA-seq transcriptomic atlas of the PFCs of patients with MDD [26]. Compared with the original annotation results from Nagy et al. [26], we referred to the recent literature [32, 39] and made more detailed annotations for neurons and astrocytes according to their developmental states. Our results showed that the proportion of astrocytes in the PFC was significantly decreased in patients with MDD. Moreover, the proportion of astrocytes changed the most dramatically compared to other cell types. In fact, many previous histopathological studies have described significant decreases in the density of astrocytes in many brain regions in MDD; however, these findings were obtained by measuring the density and size of GFAP-immunoreactive astrocyte cell bodies and GFAP mRNA expression postmortem from MDD [52–54]. To the best of our knowledge, this is the first study to confirm a decline in the proportion of astrocytes by using single-cell data. Moreover, previous studies have shown that communication between astrocytes and neurons is involved in the modulation of synaptic and network activity [55, 56]. In our study, we noticed that the greatest reduction in communication occurred between astrocytes and excitatory neurons. Moreover, it is well-known that astrocytes can be divided into diverse subtypes according to different brain areas or diseases, while the MDD-relevant subcluster classification of PFC astrocytes is largely unknown. Therefore, we further identified three astrocyte subtypes and found that C2 State-1Q astrocytes had an aberrant change at both the cell proportion and molecular level. Above all, the findings from the current study provide in vivo evidence that astrocytes, especially C2 State-1Q, may play a key role in MDD.

Autophagy varies among different cells in the CNS. In astrocytes, autophagy can be beneficial for clearing damaged proteins and maintaining function [57]. Moreover, a study showed that the autophagy-related gene ATG5 modulates astrocyte differentiation and development in the mouse cortex [58]. Furthermore, astrocyte autophagy has an impact on neuronal survival [59]. Some studies have highlighted the importance of autophagy in astrocytes in neurodegenerative disease [60–63]. However, whether impairments in autophagy in astrocytes are associated with the pathological mechanism of MDD remains largely unknown. Only one previous study on a chronic mild stress model of depression reported that the antidepressant fluoxetine can play an antidepressant role by increasing astrocyte autophagic flux [11], suggesting that astrocyte autophagy may be associated with the pathogenesis of MDD. The current study is the first to indicate that autophagy-related pathways are significantly inhibited in astrocytes in the PFC of MDD patients. Furthermore, we found that autophagy-related pathways in C2 State-1Q astrocytes were more commonly inhibited than those in other subpopulations. Interestingly, some classic autophagy-related genes, such as ATG16L and LC3A, in C2 followed a decrease in expression level along the differentiation trajectory. Based on these results, we speculated that the impaired autophagy function of C2 astrocytes may impair their homeostasis and function and may be related to the pathological mechanism of MDD. It is worth noting that mice resilient to repeated social defeat stress exhibited enhanced autophagic flux in prefrontal cortical microglia, indicating a cell-type-specific contribution of autophagy in MDD [64].

To identify some key genes that may be closely related to MDD, we integrated snRNA-seq and bulk transcriptomic data. It is well-known that bulk RNA-seq technology provides an average expression level in samples comprising different cell types, whereas snRNA-seq delivers more detailed but sparse information. Therefore, such integrative procedures could balance the strengths and weaknesses of bulk and snRNA-seq technologies when applied to identifying stable biomarkers or key genes involved in MDD. The results showed that LC3A and UBE2N were significantly downregulated in the PFCs of patients with MDD. To further validate our findings, we examined the expression levels of LC3A and UBE2N in a CUMS-induced depressed mouse model. Our results demonstrated that LC3A was significantly downregulated within the PFC region of CUMS mice, while UBE2N expression levels remained unchanged, contrasting with the downregulation observed in human postmortem brain tissues.

Members of the autophagy-related human MAP1LC3/LC3 family, including LC3A, LC3B, and LC3C, are structural proteins in the membranes of phagophores and autophagosomes [65]. Among these, LC3B is the most widely studied central autophagy protein/gene [66–68]. LC3B has been proposed as the most suitable marker for the detection of cells undergoing autophagy. Compared to LC3B, the molecular function and regulation of LC3A and LC3C have not been investigated frequently [69]. However, the conversion process of LC3A-I to LC3A-II is also considered to be closely related to autophagy initiation [70]. Some studies have reported that these genes may not be identical in their expression, function, and cellular localization [71, 72]. Recently, one study reported that the transcripts of MAP1LC3B exhibited a tendency to increase [64] but did not show a significant difference in the PFC of patients with MDD compared to healthy controls. In comparison, we observed a significant decrease in LC3A expression in the PFC region of depressed patients. These findings suggest that LC3A and LC3B may have different functions in the PFC region of MDD patients, potentially due to differential regulation by transcription factors.

Previous research from our laboratories and others has elucidated that some autophagy genes may be used as diagnostic markers of MDD [15, 73]. Therefore, we further explored whether these two genes could be used as peripheral blood diagnostic markers for MDD. RNA-seq analysis revealed that LC3A expression was significantly downregulated, whereas UBE2N expression was not significantly changed. Moreover, in a preliminary comparison, LC3B and LC3C expression was not significantly changed in patients with MDD. In addition, LC3C expression in whole blood was too low. Subsequently, we verified the downregulation of LC3A by RT-PCR in an independent larger sample cohort. ROC analysis showed a moderate discriminating power between MDD patients and controls.

This study has some limitations. First, the sample size of this study is relatively small, and although the patients had not taken any medication for at least two weeks prior to enrollment, most of them were not drug-naive, which precludes us from determining the effect of antidepressants on autophagy. Future studies should include drug-naive patients and examine changes in LC3A gene expression levels pre- and post antidepressant intervention using longitudinal analysis. Second, in this study, the connection of LC3A with autophagy is speculated. Impaired autophagy in neurons was not further investigated in this study. Further investigation is needed in future studies to explore the molecular mechanisms underlying the occurrence and development of LC3A in astrocytes and neurons in MDD. Third, lifestyle variables such as drinking, smoking, and exercise can influence the process of autophagy. Unfortunately, these variables were not recorded in our study. It will be important to consider and incorporate these variables as confounders in future studies.

In conclusion, we reported that C2 State-1Q subpopulations of astrocytes may be closely involved in MDD. In addition, we identified for the first time that autophagy pathways are commonly inhibited in astrocytes in the PFC of patients with MDD, providing new evidence that LC3A may be linked to the pathogenesis of MDD. Moreover, consistent results from PFC single-cell sequencing data, PFC bulk RNA sequencing data, whole-blood RNA sequencing data, and PCR data from whole-blood RNA showed that LC3A was significantly downregulated in MDD. Furthermore, our results suggest that LC3A may have moderate diagnostic value for MDD and is inversely correlated with the severity of depressive symptoms in MDD patients.

### Supplementary information

Supplementary Table 1

Supplementary Table 2

Supplementary Figures

## Data Availability

The expression matrix of snRNA-seq (GSE144136) and bulk RNA-seq (GSE45642, GSE53987, and GSE92538) were acquired from the Gene Expression Omnibus (GEO). All data needed to evaluate the conclusions in the paper are present in the paper and/or the Supplementary Materials. Additional data are available from authors upon request.

## Code availability

The code used for the analysis in this study is available upon request.
