# Supplementary material for: Does decreased autophagy and dysregulation of LC3A in astrocytes play a role in major depressive disorder?
Source: Transl Psychiatry. 2023 Nov 25;13:362. doi: 10.1038/s41398-023-02665-2 (PMC10673997; doi:10.1038/s41398-023-02665-2)
Supplement: Supplementary file 1 — Supplementary Table 1 [file 41398_2023_2665_MOESM1_ESM.docx]

**Supplementary Table 1. Demographic and clinical characteristics of MDD patients and controls for RNA-seq.**

| **Variable** | **MDD**  **(n=19)** | **Healthy**  **(n=17)** | **Statistic** | **P Value** |
| --- | --- | --- | --- | --- |
| Age (mean±SD, year) | 37.16±13.19 | 36.12±11.16 | t=-0.254 | 0.801 |
| Sex |  |  | χ^2^=0.385 | 0.535 |
| Female | 12 | 9 |  |  |
| Male | 7 | 8 |  |  |
| BMI | 19.83±2.77 | 19.91±2.99 | t=0.089 | 0.929 |
| HAMD-17 | 29.42±10.67 |  |  |  |
| Duration of current episode (month) | 4.29±10.94 |  |  |  |
| Duration of MDD (month) | 30.74±41.81 |  |  |  |
| Family history | 6 |  |  |  |
| Medication history |  |  |  |  |
| Drug naïve | 4 |  |  |  |
| Used SSRIs | 7 |  |  |  |
| Used SNRIs | 6 |  |  |  |
| Used NaSSAs | 2 |  |  |  |

Abbreviations: MDD, major depressive disorder; BMI, body mass index; HAMD-17, 17-item Hamilton Depression Rating Scale; SSRIs, Selective serotonin reuptake inhibitors; SNRIs, Selective norepinephrine reuptake inhibitors; NaSSAs, Noradrenergic and specific serotonergic antidepressants.
