# Supplementary material for: Does decreased autophagy and dysregulation of LC3A in astrocytes play a role in major depressive disorder?
Source: Transl Psychiatry. 2023 Nov 25;13:362. doi: 10.1038/s41398-023-02665-2 (PMC10673997; doi:10.1038/s41398-023-02665-2)
Supplement: Supplementary file 2 — Supplementary Table 2 [file 41398_2023_2665_MOESM2_ESM.docx]

**Supplementary Table 2. Demographic and clinical characteristics of MDD patients and controls for qPCR**

| **Variable** | **MDD**  **(n=33)** | **Healthy**  **(n=32)** | **Statistic** | **P Value** |
| --- | --- | --- | --- | --- |
| Age (mean±SD, year) | 35.12±14.564 | 26.84±9.098 | z=-2.145 | 0.032 |
| Sex |  |  | χ^2^=0.905 | 0.341 |
| Female | 27 | 23 |  |  |
| Male | 6 | 9 |  |  |
| BMI | 21.83±3.244 | 22.09±3.527 | z=-0.203 | 0.839 |
| HAMD-17, mean±SD | 33.06±10.028 |  |  |  |
| Duration of current episode (month) | 8.09±13.545 |  |  |  |
| Duration of MDD (month) | 34.09±78.592 |  |  |  |
| Family history | 7 |  |  |  |
| Medication history |  |  |  |  |
| Drug naïve | 5 |  |  |  |
| Used SSRI | 19 |  |  |  |
| Used SNRI | 6 |  |  |  |
| Used NaSSAs | 3 |  |  |  |

Abbreviations: MDD, major depressive disorder; BMI, body mass index; HAMD-17, 17-item Hamilton Depression Rating Scale; SSRIs, Selective serotonin reuptake inhibitors; SNRIs, Selective norepinephrine reuptake inhibitors; NaSSAs, Noradrenergic and specific serotonergic antidepressants.
