# Supplementary material for: Does decreased autophagy and dysregulation of LC3A in astrocytes play a role in major depressive disorder?
Source: Transl Psychiatry. 2023 Nov 25;13:362. doi: 10.1038/s41398-023-02665-2 (PMC10673997; doi:10.1038/s41398-023-02665-2)
Supplement: Supplementary file 3 — Supplementary Figures [file 41398_2023_2665_MOESM3_ESM.docx]

**Supplemental Figures**

**Figure S1**


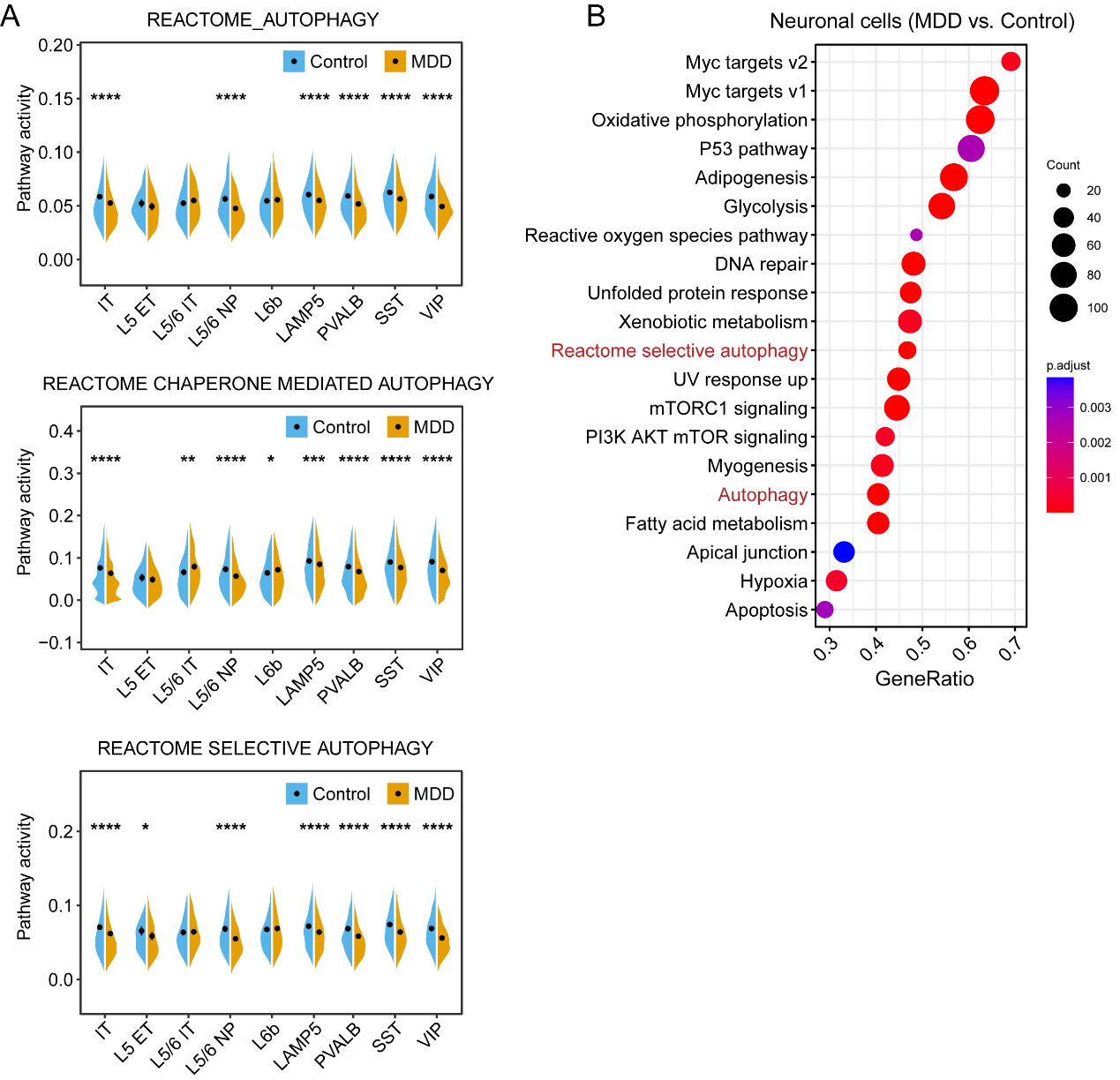


Figure S1. The association between neuronal cells and autophagy pathways in the prefrontal cortex (PFC) of major depressive disorder (MDD). A. AUCell analysis showing the activity of different autophagy pathways of neuronal cells in MDD. B. Top 20 enriched pathways identified by GSEA in neurons comparing subjects with MDD to control subjects.

**Figure S2**


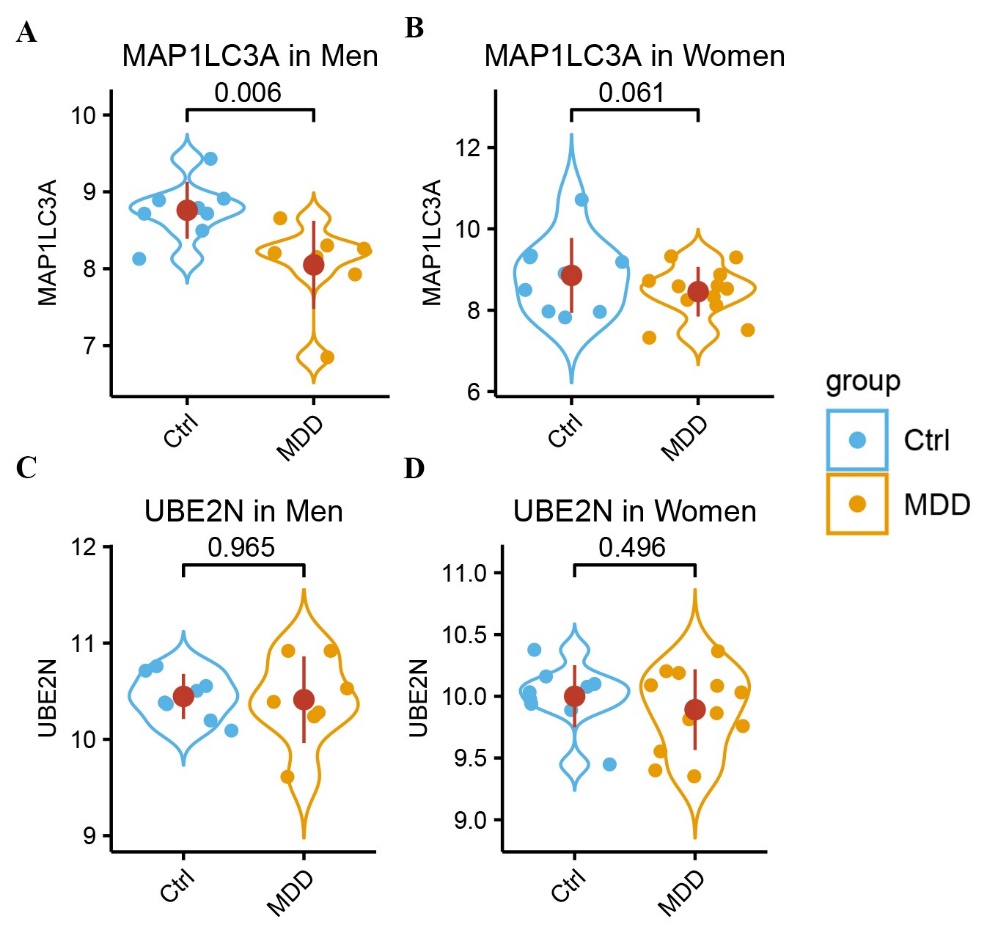


Figure S2. The expression levels of LC3A and UBE2N in males and females in RNA-seq data (A-D).

**Figure S3**


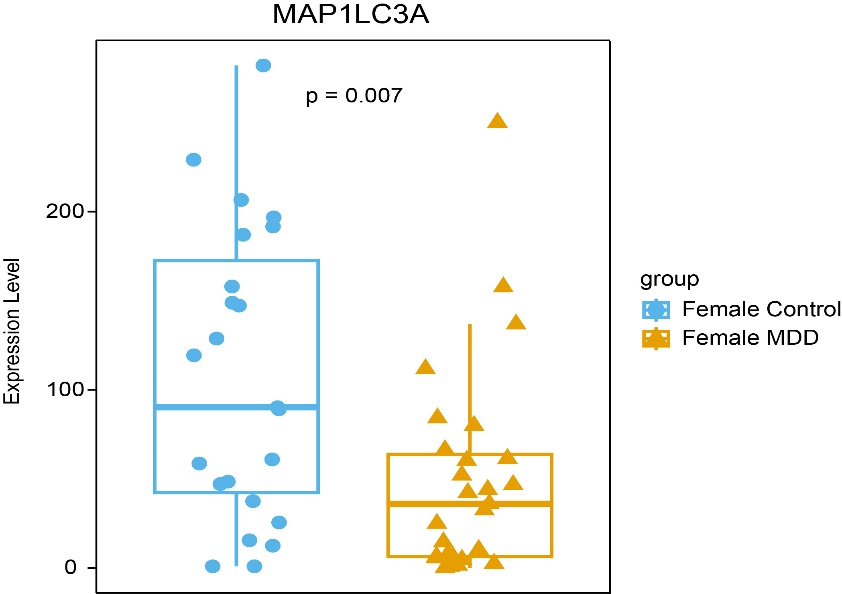


Figure S3. LC3A expression among female MDD subjects compared to healthy female controls in PCR data.

**Figure S4**


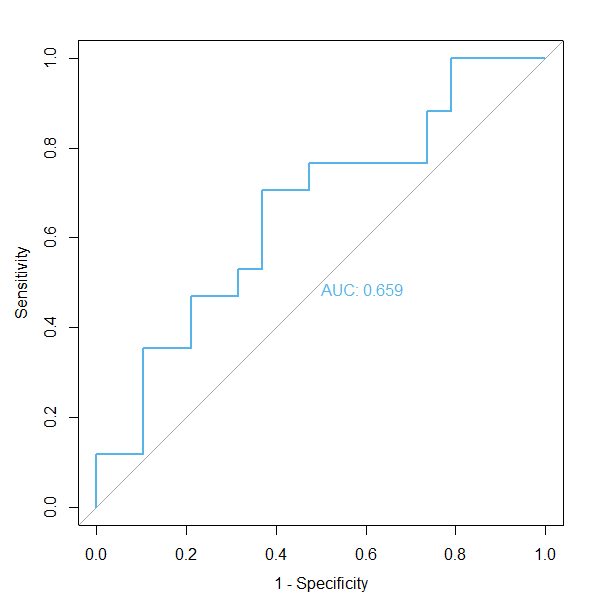


Figure S4. ROC analysis for LC3A between patients with MDD and healthy controls in RNA-seq data.
